# Preparation and Properties of PA10T/PPO Blends Compatibilized with SEBS-g-MAH

**DOI:** 10.3390/polym16111598

**Published:** 2024-06-05

**Authors:** Housheng Xia, Zhen Jiang, Jiaxiang Tang, Jiao Tang, Jianping Zhou, Zize Yang, Rongbo Zheng, Junfeng Niu

**Affiliations:** 1 School of Biological and Chemical Engineering, Zhejiang University of Science and Technology, Hangzhou 310023, China; 2 Zhejiang Xianghe Railway Fastener Research Institute, Zhejiang Tiantai Xianghe Industrial Co., Ltd., Taizhou 317200, China

**Keywords:** poly(decamethylene terephthalamide), poly(phenylene oxide), compatibilizer, SEBS-g-MAH, dielectric property

## Abstract

Plant-derived PA10T is regarded as one of the most promising semi-aromatic polyamides; however, shortcomings, including low dimensional accuracy, high moisture absorption, and relatively high dielectric constant and loss, have impeded its extensive utilization. Polymer blending is a versatile and cost-effective method to fabricate new polymeric materials with excellent comprehensive performance. In this study, various ratios of PA10T/PPO blends were fabricated via melt blending with the addition of a SEBS-g-MAH compatibilizer. Molau test and scanning electron microscopy (SEM) were employed to study the influence of SEBS-g-MAH on the compatibility of PA10T and PPO. These studies indicated that SEBS-g-MAH effectively refines the domain size of the dispersed PPO phase and improves the dispersion stability of PPO particles within a hexafluoroisopropanol solvent. This result was attributed to the in situ formation of the SEBS-g-PA10T copolymer, which serves as a compatibilizer. Differential scanning calorimetry (DSC) and thermogravimetric analysis (TGA) results showed that the melting–crystallization behavior and thermal stability of blends closely resembled that of pure PA10T. Dynamic mechanical analysis (DMA) revealed that as the PPO content increased, there was a decrease in the glass transition temperature and storage modulus of PA10T. The water absorption rate, injection molding shrinkage, dielectric properties, and mechanical strength of blends were also systematically investigated. As the PPO content increased from 10% to 40%, the dielectric loss at 2.5 GHz decreased significantly from 0.00866 to 0.00572, while the notched Izod impact strength increased from 7.9 kJ/m^2^ to 13.7 kJ/m^2^.

## 1. Introduction

With the development of next-generation communication technologies, polymer materials with a low dielectric constant (D_k_) and low dielectric loss (D_f_) are urgently needed for high-speed communication devices [1]. Meanwhile, they are required to meet various other criteria, including moisture uptake, high-temperature resistance, mechanical properties, dimensional accuracy, and so on [2,3]. As part of high-performance engineering plastics, polyphthalamides (PPA) are mainly synthesized through polycondensation reactions between aliphatic diamines and aromatic acids. They inherit the advantages of the processing ability of aliphatic polyamides and the heat resistance of wholly aromatic polyamides, thereby leading to their wide applications in the automobile, electrical, military, and aerospace industries [4]. The overwhelming majority of PPA homopolymers cannot be melt-processed because their melting points (Mp) are even higher than their decomposition temperatures; consequently, commercially available PPAs typically exist as copolymers with suitable melting points (Mp) that can be deliberately reduced by incorporating specific copolymerization components. Unfortunately, in these PPA copolymers, crystallinity is compromised, rendering the attainment of injection-molded parts with high crystallinity challenging, particularly under rapid solidification conditions. Furthermore, the common copolymerization components, like PA66 and PA6, can markedly elevate the moisture absorption and D_f_ of homopolymer PPA.

Increasing the carbon-chain length of the aliphatic diamine represents another efficacious strategy for reducing Mp of PPA owing to the increase in the flexible -CH_2_- segment and the decrease in hydrogen bond content. Therefore, a series of homopolymer semi-aromatic polyamides with melting points ranging from 285 °C to 316 °C, including PA9T, PA10T, and PA12T, have been effectively synthesized by the reaction of terephthalic acid with 1,9-nonanediamine, 1,10-decanediamine, and 1,12-dodecanediamine, respectively [5,6]. Notably, 1,10-decanediamine is derived from renewable castor beans, thus rendering PA10T a subject of considerable interest in both industrial and academic domains [7]; however, in contrast to other high-performance engineering plastics like polyphenylene sulfide (PPS), PA10T is not considered a favorable choice for next-generation communication applications due to its relatively high molding shrinkage, moisture absorption, D_k_, and D_f_ [8,9].

Recently, researchers discovered that the integration of fluorinated groups into PPA brings significant benefits in enhancing electrical and dielectric performance [10,11]. This improvement is attributed to the low polarizability and diminutive dipole moment of the C–F bond, combined with an increased free volume. Ge et al. [12] synthesized fluorinated PPAs by polymerizing various aromatic diamines with two novel monomers, namely, 5-(3,5-bistrifluoromethyl-phenoxy) isophthaloyl dichloride and 5-(4-trifluoromethylphenoxy) isophthaloyl dichloride. The D_k_ and D_f_ values of the fluorinated PPAs at 1 MHz can be reduced to as low as 3.24 and 2.75 × 10^−3^, respectively. An additional strategy for the development of low-k PPA involves integrating porous materials. As a representative, polyhedral oligomeric silsesquioxane (POSS) consists of a cage-like skeleton and efficiently reduces the k value of polymeric composites [13,14]. Luo et al. [15] developed a reversibly cross-linked composite by incorporating a maleimide-functionalized POSS to PPA resin containing furan groups. The porous characteristics of POSS, together with the restricted movement of polymer chains due to cross-linking, result in a notable reduction in the D_k_ and D_f_ of the composites. However, fluoro-containing monomers and POSS are not widely available as commodity chemicals, thus hindering the large-scale application of these modified PPAs as next-generation communication materials in the short term.

Blending commercially available polymers offers a more cost-effective strategy for creating new materials that satisfy specific market demands, compared to the development of a new type of resin [16,17]. Poly(2,6-dimethyl-1,4-phenylene oxide) (PPO) is an amorphous engineering plastic with a high glass-transition temperature. It has favorable characteristics including low D_k_ and D_f_, water resistance, and good dimensional stability. Consequently, the development of PA/PPO blends has been the subject of extensive investigation [18]. To date, various blends of aliphatic PA and PPO have been developed by using effective compatibilizers [19,20,21]. Unfortunately, aliphatic PA containing a high proportion of polar amide groups exhibits elevated D_k_ and D_f_, which is an inherent drawback in the development of communication materials. Modified PPAs have better thermal-resistance properties, and lower D_k_ and D_f_, rendering them potential candidates for developing PA/PPO blends with superior properties [22].

Recently, we developed PA10T/PPO blends using a series of copolymer-type compatibilizers [23]. The results indicated that the reactive copolymer comprising styrene (St) and glycidyl methacrylate (GMA) effectively refined the domain size of the dispersed PPO phase and enhanced the mechanical strength of PA10T/PPO blends. However, the poly (St-co-GMA) compatibilizer is not accessible to plastic compounding companies and exhibits relatively high D_f_ due to the presence of both ester and epoxide groups in GMA components; therefore, there is still a need to develop high-performance PPA/PPO blends with low D_k_ and D_f_ by choosing more suitable compatibilizers.

The styrene–ethylene–butadiene–styrene block copolymer grafted with maleic anhydride (SEBS-g-MAH) is available commercially and has been extensively used as both a compatibilizer and a toughening agent for PA/PPO blends [24]. Due to its saturated EB blocks, SEBS-g-MAH exhibits enough thermal stability for high-temperature processing. Moreover, it has low D_f_ because its MAH grafting ratio is usually less than 2.0% [25]. Therefore, SEBS-g-MAH was chosen to improve the compatibility between PA10T and PPO components. The micro-morphology, mechanical, thermal, and dielectric properties of the prepared PA10T/PPO blends were studied. The results suggested that SEBS-g-MAH refined the micromorphology of PPO. Furthermore, the addition of PPO components substantially enhanced the dielectric properties of PA10T/PPO blends, albeit with a slight compromise in mechanical properties. The developed PA10T/PPO blends, possessing outstanding comprehensive properties, are envisioned to hold promising applications in next-generation communication fields.

## 2. Materials and Methods

### 2.1. Materials

Poly(decamethylene terephthalamide) (PA10T, P-8013) with a relative viscosity of 2.0~2.2 was purchased from GCL New Materials Co., Ltd., Jining, China. Poly(2,6-dimethyl-1,4-phenylene oxide) (PPO, LXR035) was supplied by Bluestar New Chemical Materials Co., Ltd., Ruicheng Branch, Yuncheng, China. SEBS-MAH (Kraton FG1901X, Kraton Polymer Co., Ltd., Houston, TX, USA) is a block copolymer with 29% styrene and 71% hydrogenated butadiene, in which the hydrogenated butadiene block is grafted with 1.4~2.0% maleic anhydride.

### 2.2. Preparation of PA10T/PPO Blends

All materials, excluding SEBS-g-MAH, underwent drying in air-circulating ovens at 120 °C for 8 h before processing. Blends comprising PA10T and PPO were prepared by melt-compounding using a twin-screw extruder (SHJ-35-40D, Nanjing Keya Extrusion Equipment Co., Ltd., Nanjing, China). The screw speed was set at 300 rpm. The barrel temperatures were set at 310 °C/315 °C/320 °C/325 °C/330 °C from feed inlet to die. Subsequently, the extruded pellets were dried and molded into test specimens using an injection machine (Haitian PL860, Haitian Plastics Machinery Co., Ltd., Ningbo, China) at a mold temperature of 150 °C. The processing temperatures from the hopper to nozzle were set at 310 °C, 320 °C, 330 °C, and 330 °C, respectively. To systematically study the effect of PPO content on the various properties of the blends, compatibilized PA10T/PPO blends with a PPO content interval of 10–40% were prepared. The blends, containing PPO at 10%, 20%, 30%, and 40% (excluding the compatibilizer), with a consistent weight ratio of SEBS-g-MAH (PPO:SEBS-g-MAH = 5:1), were formulated and labeled as B1, B2, B3, and B4, respectively. Pure PA10T specimens were also prepared with the same process for comparison purposes. A summary of PA10T and the prepared PA10T/PPO blends is presented in Table 1.

### 2.3. Characterization

Attenuated total reflectance Fourier-transform infrared spectra (ATR/FT-IR) were obtained from a Thermo Nicolet 6700 FT-IR spectrometer. The settings included 64 co-added scans per sample, a spectral resolution of 2 cm^−1^, and a wavenumber range from 4000 to 650 cm^−1^.

The Molau test was conducted by dissolving 0.5 g of the samples in 20 mL of hexafluoroisopropanol (HFIP) with intermittent ultrasonic vibration for 24 h.

The melt flow index (MFI, 330 °C/1.2 kg) was investigated using a melt flow index tester (ZRZ1402, Shenzhen Sansi Co., Ltd., Shenzhen, China).

The morphological properties of blends were analyzed using scanning electron microscopy (SEM, Zeiss-Gemini 300, Carl Zeiss AG Co., Ltd., Oberkochen, Germany) with an accelerating voltage of 20 kV. The specimens were broken at liquid nitrogen and then etched in THF for 12 h. The purpose was to dissolve the dispersed PPO phase. Before SEM observations, all samples were dried entirely at 80 °C for 8 h with a vacuum oven and then coated with a thin layer of Au.

The melting–crystallization behavior of blends was analyzed by differential scanning calorimetry (DSC, Q2000) from TA Instruments. The measurements were conducted from 30 °C to 330 °C under the N_2_ atmosphere (50 mL/min), utilizing a consistent rate of 10 °C/min for heating and cooling processes.

Thermogravimetric analyses (TGA, TA instrument/Q500) were conducted from 30 °C to 750 °C at a 10 °C/min heating rate in the N_2_ atmosphere.

Dynamic mechanical properties of blends were studied by a dynamic mechanical analyzer (DMA7E, Perkin Elmer Inc. Co., Ltd., Waltham, MA, USA). The injection molded samples were mechanically cut into rectangular strips with dimensions of 35 mm × 6 mm × 2 mm. The test was conducted from room temperature to 280 °C with a heating rate of 5 °C/min, at a frequency of 1 Hz, and with a stretching ratio of 0.1%.

The pure PA10T and blends were injected into shapes measuring 80 mm × 80 mm × 2 mm to determine dielectric constants and dielectric loss on a vector network analyzer (Agilent E5071C, Agilent Co., Ltd., Santa Clara, CA, USA) at a resonant frequency of 2.5 GHz using the cavity resonator method.

Shrinkage of the square specimens with sizes of 80 mm × 80 mm × 2 mm was evaluated 48 h after injection molding [26]. Shrinkage values were calculated by the following equation.

(1) $$Shrinkage\;(\%)=\frac{l_{mold}-l_{part}}{l_{mold}}\;\times \;100\;\;\;\;\;$$

where *l_mold_* represents the length of the mold and *l_part_* represents the average length of the square specimens. The average values were determined by 5 samples for each composition.

The water absorption was tested in compliance with the ISO 62 standard [27]. The tensile specimens were immersed in deionized H_2_O. The weight of specimens was measured every day using an analytical weighing balance (Mettler-Toledo AL104) until their values remained constant. Finally, water absorption rates were calculated according to Equation (2) [28]:

(2) $$Water\;absorption\;(\%)=\frac{W_{final}-W_{intial}}{W_{intial}}\;\times \;100$$

where *W_intial_* represents the initial weight of the dry specimen before soaking, and *W_final_* represents the final weight of wet specimens after water uptakes reach saturation. Saturation with water was observed in all samples within a period of 90 to 120 days.

Tensile and flexural properties were tested using a Universal Testing Machine (UTM-4304, Shenzhen Sansi Co., Ltd., Shenzhen, China). The tests were conducted following ISO 527 [29] for tensile properties and ISO 178 [30] for flexural properties, with testing speeds set at 10 mm/min and 2 mm/min, respectively. Impact tests were conducted on an impact tester (ZBC1251-2, Sansi, China) following the ISO 180 standard [31].

## 3. Results

### 3.1. Compatiblizing Effect of SEBS-g-MAH on PA10T/PPO Blend

**Figure 1:**
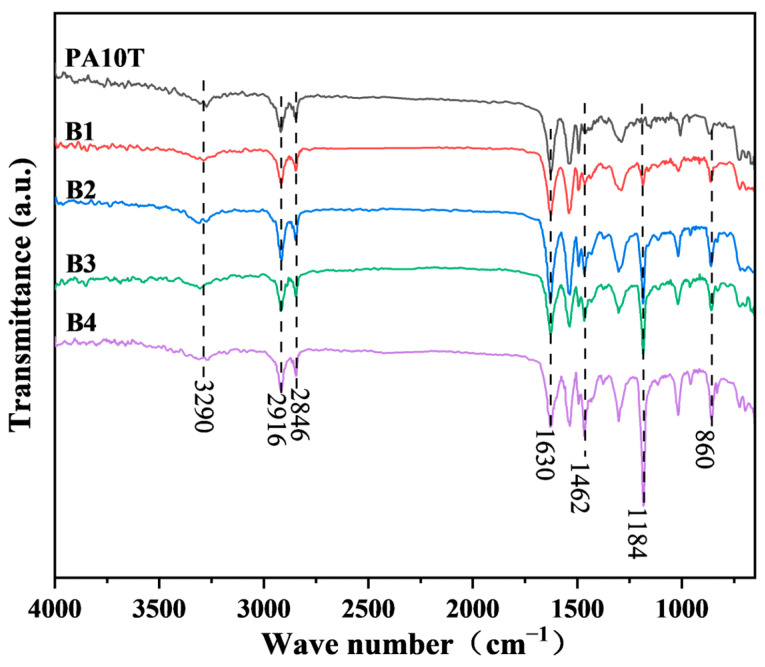
ATR/FT-IR spectra of pure PA10T and the PA10T/PPO blends.

Figure 1 presents the ATR/FT-IR spectra of pure PA10T and the blends. Peaks around 3290 cm^−1^ and 2916 cm^−1^ are assigned to the N-H and C-H stretching vibrations, respectively. These peaks represent characteristic absorptions of PA10T. The peak around 1628 cm^−1^ is due to the stretching vibrations of the C=O group originating from the amide group of PA10T [32]. On the other hand, peaks at 1462 cm^−1^ and 1184 cm^−1^ are characteristic peaks of PPO [33]. The intensity of these peaks increases proportionally with the increase of PPO content from 10% to 40%. The ATR/FT-IR results demonstrated the successful preparation of the PA10T/PPO blends with the expected composition. Notably, no significant carbonyl resonance from the MAH groups in the SEBS-g-MAH compatibilizer (1858 cm^−1^ and 1772 cm^−1^) was observed [34], probably due to the complete reaction of MAH with PA10T, or its content being below the limit of FT-IR detection.

**Figure 2:**
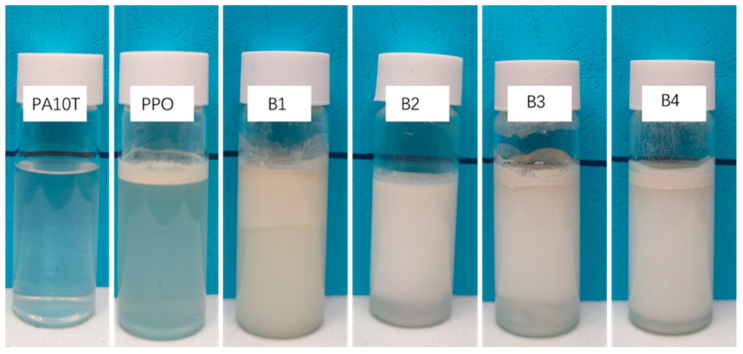
Molau test solutions in HFIP for pure PA10T, PPO, and the PA10T/PPO blends.

**Figure 3:**
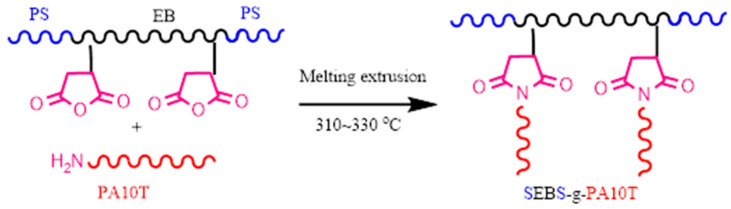
A possible mechanism of the formation of SEBS-g-PA10T macromolecules.

The Molau test is a practical approach for characterizing the compatibility of polymer blends. This analysis relies on the disparity in polymer solubility, enabling investigation into the production of grafted copolymers within reactive blends [35]; therefore, the Molau test was used to assess whether a grafted copolymer forms between PA10T and SEBS-g-MAH. As shown in Figure 2, pure PA10T resin is wholly dissolved in hexafluoroisopropanol (HFIP), and forms a transparent solution. Conversely, pure PPO resin remains insoluble, with PPO powder aggregating in suspension within the upper portion of the same test tube. Each of the PA10T/PPO blends containing the SEBS-g-MAH compatibilizer forms a milky suspension in HFIP media, suggesting the presence of a substance capable of mitigating the agglomeration and flotation of PPO domains. It is speculated that an amidation reaction occurred between the amino end-groups of PA10T and the MAH side-groups of SEBS-g-MAH during the melt blending (Figure 3). In other words, the in situ formation of “comb-like” SEBS-g-PA10T macromolecules serves as an emulsifying agent, effectively stabilizing the PPO particles within the PA10T/PPO/HFIP suspension.

**Figure 4:**
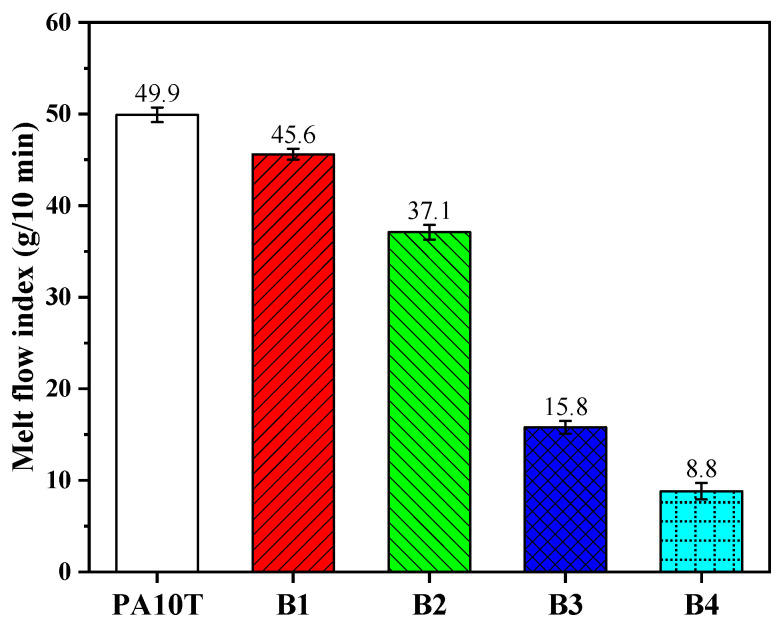
Melt flow index of pure PA10T and the PA10T/PPO blends.

Figure 4 illustrates the melt flow index (MFI) of pure PA10T and the blends. The MFI values decreased with increasing PPO and SEBS-g-MAH content. The MFI of B1 is 45.6 g/10 min, slightly lower than that of pure PA10T (49.9 g/10 min). The MFI of B2 decreases steadily to 37.1 g/10 min but the MFI values of B3 and B4 decrease sharply to 15.8 g/10 min and 8.8 g/10 min, respectively. These variations are primarily related to the intrinsic viscosity of PPO and SEBS-g-MAH. As reported in our prior research, the selected PPO resin—a commercial product with low viscosity—has positively influenced the MFI value of the un-compatibilized PA10T/PPO blend. The physical influence of SEBS-g-MAH is very low because of its low content. Therefore, the chain-extending effect of SEBS-g-MAH is presumed to be the main reason for the decrease in MFI. The chemical interaction between SEBS-g-MAH and PA10T leads to the production of SEBS-g-PA10T supermacromolecules, which can significantly increase the melt viscosity of blends.

**Figure 5:**
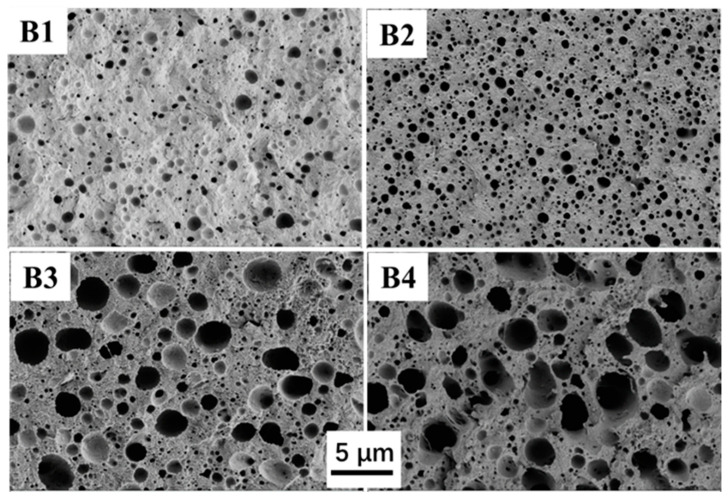
SEM micrographs of the tetrahydrofuran-etched fracture surface of the compatibilized PA10T/PPO blends containing 10%, 20%, 30%, and 40% PPO.

Figure 5 shows the SEM micrographs of the tetrahydrofuran (THF)-etched fracture surfaces of the blends. The blends exhibit numerous randomly dispersed voids. These voids indicate the dispersed morphology of PPO, which was selectively dissolved by THF solvent [36]. The average diameters of the dispersed PPO phase in the B1, B2, B3, and B4 samples are 0.52 μm, 0.47 μm, 1.59 μm, and 1.97 um, respectively. The values of all blends are notably lower compared to the un-compatibilized PA10T/PPO blend (*w*/*w* = 80/20, 2.1 μm) outlined in our prior research [23]. Although the B1 sample has the lowest PPO content, its PPO domain size is larger than that of blend B2 due to insufficient SEBS-g-MAH (~2%) to minimize the interfacial tension between PA10T and PPO. These results indicate that SEBS-g-MAH is an effective compatibilizer but its efficiency is relatively low. The low efficiency may stem from several factors: (1) the MAH grafting ratio of the present SEBS-g-MAH is only 2%; (2) the hydrogenated butadiene block is incompatible with both PA10T and PPO, yet its proportion reaches 71%; (3) the grafted MAH groups can react with the amide (-NH_2_) groups at one end of PA10T chain but are unreactive to carbonyl (-COOH) groups at the opposite end. With the simultaneous rise in PPO/SEBS-g-MAH content, the average diameter of PPO domains in B3 increases significantly, while certain PPO domains in B4 aggregate marginally into irregular particles, typical of phase-separation phenomena in incompatible blends. Furthermore, the interfaces between PPO and PA10T in blends B3 and B4 appear coarse, indicating good interfacial adhesion between the two phases. This result implies that SEBS-g-MAH exerts a satisfactory compatibilizing effect on the PA10T/PPO blend when its content reaches a sufficient level.

### 3.2. Thermal Properties

**Figure 6:**
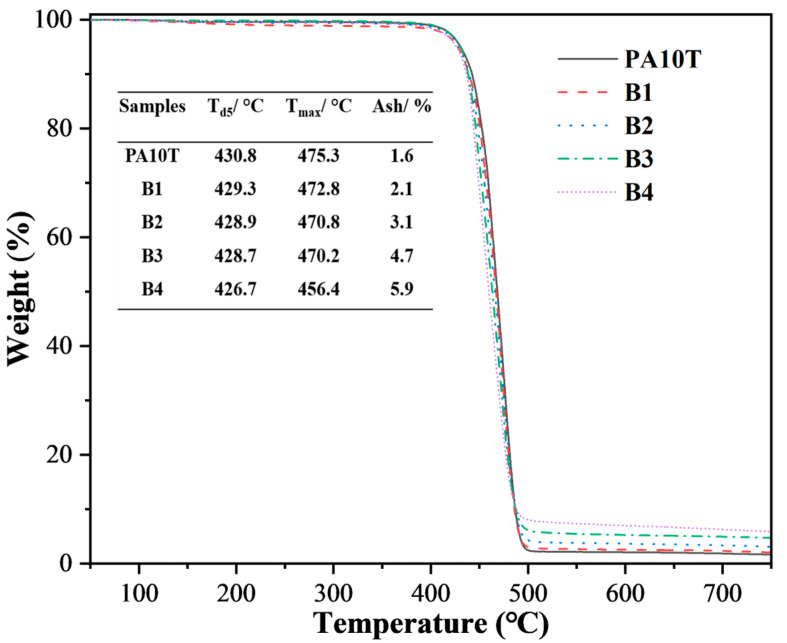
TGA curves of pure PA10T and the PA10T/PPO blends.

TGA curves of both pure PA10T and the blends under the N_2_ atmosphere are shown in Figure 6. Each sample exhibits comparable trends in weight loss and displays outstanding thermal stability. The temperature at 5% weight loss (T_d5_) for pure PA10T is 430.8 °C, and its maximum degradation temperature (T_max_) reaches 475.3 °C. The T_d5_ and T_max_ for PA10T/PPO blends range from 426.7 °C to 429.3 °C and 456.4 °C to 472.8 °C, respectively, and are marginally below the values observed in pure PA10T. Adding PPO decreases the heat resistance of PA10T, a trend consistent with our prior research [28]. This phenomenon may be caused by a residual catalyst in the PPO resin, which could promote the degradation of the macromolecular chains in PA10T [37]. The final ash residues of PA10T/PPO blends at 750 °C increase significantly with increasing PPO content, indicating that introducing PPO resin could improve the char-forming capability of the polymer matrix. Indeed, PPO resin is easily carbonized at elevated temperatures; thus, it is widely employed as a carbon-forming agent in the fabrication of halogen-free flame-retardant composites [38].

**Figure 7:**
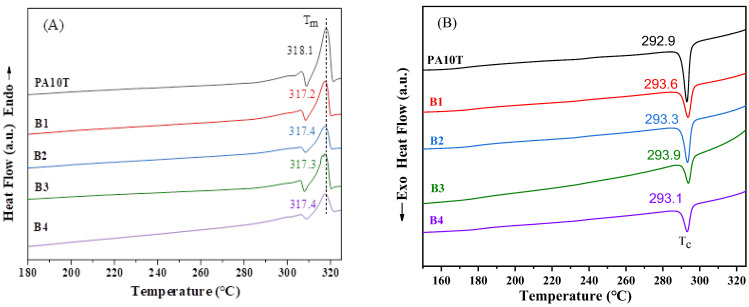
DSC curves of pure PA10T and the PA10T/PPO blends: (**A**) second heating run; and (**B**) cooling from the melt.

The melting and crystallization behavior of pure PA10T and the blends were analyzed by DSC. In the second heating curve (Figure 7A), double melting endotherms are observed, which can be attributed to the melting–recrystallization–melting mechanism as proposed by Li [39]. The shoulder peaks appear at lower temperatures, indicating the presence of metastable crystals in all samples, corresponding to the melting of defective PA10T crystals. The PA10T/PPO blends exhibit comparable melting peaks (T_m_ = 317.2 °C~317.4 °C), slightly lower than pure PA10T (T_m_ = 318.1 °C). As shown in Figure 7B, the crystallization temperatures of the blends are slightly higher than the value of pure PA10T (292.9 °C). These results suggest that the inclusion of PPO and SEBS-g-MAH components exerts a nucleating effect on the crystallization of PA10T, facilitating the formation of fine PA10T crystallites. Notably, the glass-transition signals of both PA10T and PPO are indistinct in all DSC curves owing to the low PPO content and the limited amount of PA10T in the amorphous state.

**Figure 8:**
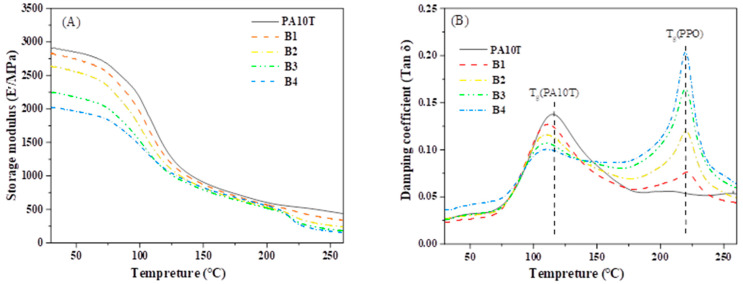
(**A**) Storage modulus and (**B**) damping coefficient of pure PA10T and the PA10T/PPO blends.

Figure 8 shows the storage modulus (E′) and damping coefficient (tan δ) of pure PA10T and the blends. The E′ gradually decreased with increasing PPO/SEBS-g-MAH loading over a wide temperature range; it shows almost the same trend as the flexural modulus obtained from the bending strength test conducted at room temperature. This phenomenon may arise from the inherent characteristics of PA10T resin, such as its rigid benzene rings, robust hydrogen bonding interactions, and high crystallinity, contributing to its exceptional resistance to dynamic deformation. PPO resin is an amorphous polymer with a soft ether group. In addition, the SEBS-g-MAH component has been widely used as a well-known elastomer to produce engineering plastic with high toughness but low stiffness [40]. Consequently, the PA10T/PPO blends incorporating PPO and SEBS-g-MAH exhibit a lower storage modulus (E′) than pure PA10T. The temperature at which the tan δ curve reaches its maximum peak is regarded as the glass transition temperature (T_g_). The uncrystallized PA10T phase in all samples has a T_g_ around 115 °C, and the value decreases slightly with increasing PPO/SEBS-g-MAH content. It is hypothesized that a fraction of the PPO or SEBS-g-MAH chains may integrate into the uncrystallized PA10T, thereby attenuating the hydrogen bonds between adjacent PA10T chains. Therefore, some PA10T segments bound by hydrogen-bonding interactions can be released at lower temperatures and have a lower T_g_ value. The PA10T/PPO blends show a similar T_g_ around 221.7 °C for the PPO phase because the compatible PPO/SEBS-g-MAH phase has a fixed ratio (PPO:SEBS-g-MAH = 5:1). The peak intensity increases proportionally with the PPO/SEBS-g-MAH content, demonstrating the successful preparation of blends with the expected composition.

### 3.3. Water Absorption Properties

**Figure 9:**
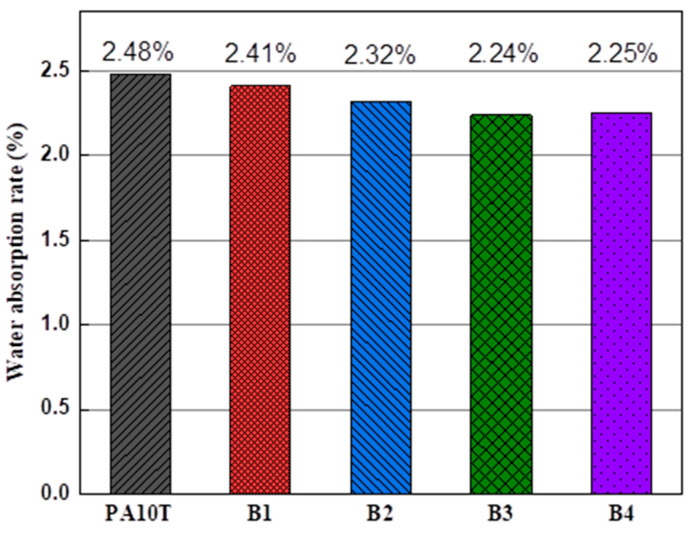
Water absorption rates of pure PA10T and the PA10T/PPO blends.

Figure 9 displays the measured water absorption rates (W) of pure PA10T and the blends. The W value of pure PA10T is about 2.48%, attributed to its inherent water-absorption capacity stemming from the polar amide groups and the amino/carboxyl end groups. Its W value is lower compared to aliphatic polyamides or semi-aromatic copolyamides containing aliphatic polyamide segments (e.g., PA6T/PA6, PA6T/PA66), owing to the lower proportion of polar groups in PA10T. The W values in blends B1 to B3 exhibit a continuous decrease with increasing PPO content due to the inherently low water absorption capacity of PPO (~0.2%) [19]. In general, the PA10T/PPO blends demonstrate promising potential for applications in environments characterized by high humidity or moisture. Nevertheless, the measured water absorption values exceed expectations, with a notably pronounced deviation observed when the PPO content increases beyond 30% to 40%. This phenomenon is likely attributable to the presence of micro-pores in the injection-molded samples. These micropores are formed due to several factors: (1) the propensity of unstable substances such as monomers or oligomers in PPO and SEBS-g-MAH to decompose at processing temperatures; (2) the addition of high-viscosity PPO resulting in elevated shear heat, thereby expediting the decomposition of unstable substances during extrusion and injection processes; (3) insufficient degassing caused by both the rapid crystallization rate of PA10T and the high melt viscosity of the PA10T/PPO blends. In fact, during the processing of samples with high PPO content, significant smoke and unpleasant odors were observed emanating from the barrel vent. The observed experimental phenomenon can be attributed to the temperature rise caused by high-speed screw shearing [41]. The shear force imparted to the polymer melt by the screw is directly proportional to the dynamic viscosity. Consequently, blends with higher PPO content exhibit higher melt viscosity and experience stronger shearing forces. This can result in localized temperatures exceeding the degradation temperature of PA10T/PPO blends, despite their high thermal resistance in a nitrogen atmosphere as indicated by TGA curves. Nevertheless, the thermal degradation observed in PA10T/PPO blends with 10–40% PPO content remains acceptable for potential scale-up production.

### 3.4. Dimensional Accuracy

**Figure 10:**
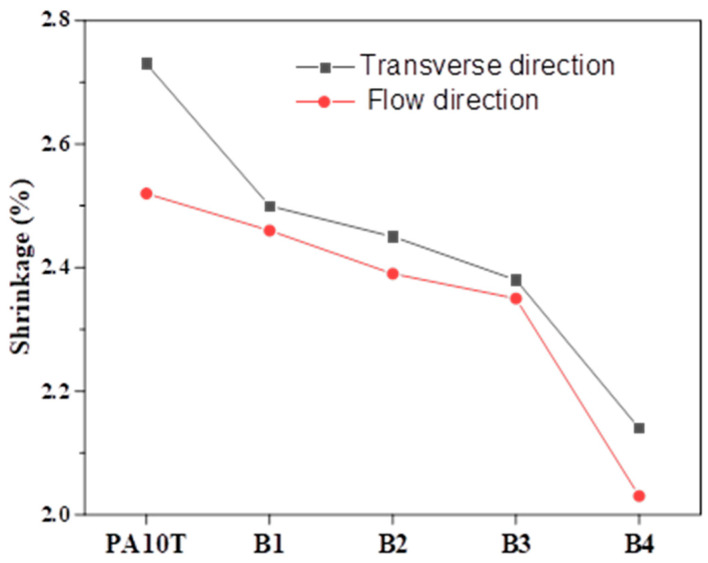
Shrinkage rates of pure PA10T and the PA10T/PPO blends.

Figure 10 shows the shrinkage rates (S) of pure PA10T and the blends, calculated from the dimensional data of the metal mold and the injection molding plates. The shrinkage rates in the transverse direction (ST) for all samples are significantly lower than those in the flow direction (SF). This difference can be attributed to the orientation behavior of the polymer chains and crystals during the injection molding process. Pure PA10T exhibits the highest ST and SF values owing to its robust crystallization ability, a trait associated with its regular chain structure, and the favorable molecular mobility of the 1,10-decane-diamine monomer. It should be highlighted that the significant crystallinity of PA10T may result in unanticipated shrinkage and internal stress, particularly in products with thick walls. These issues can negatively impact the material’s dimensional precision, flexibility, and service stability. Compared to pure PA10T, the PA10T/PPO blends have a lower S value and gradually decrease as the PPO content increases; this result is due to the rigidity of benzene rings in the PPO mainchain and the high T_g_ of the amorphous PPO. With a PPO content of 40%, ST and SF values of the B4 sample rapidly decrease to 2.2% and 2.0%, respectively; this phenomenon arises from the tendency of PPO domains with high content to aggregate, forming a partially continuous PPO phase, which contributes more to reducing the shrinkage of blends than that of a dispersed PPO phase.

### 3.5. Dielectric Properties

**Figure 11:**
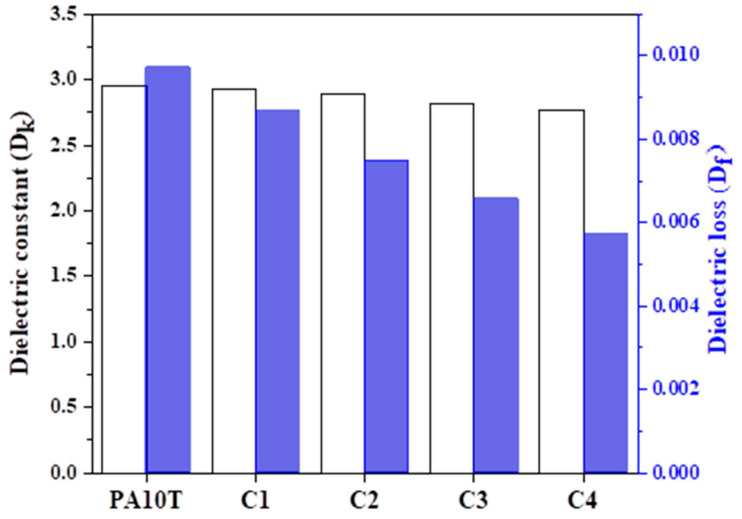
The D_k_ and D_f_ values of pure PA10T and the PA10T/PPO blends.

Figure 11 shows the D_k_ and D_f_ of pure PA10T and the blends at a frequency of 2.5 GH. All samples exhibit remarkably low D_k_ ranging from 2.76 to 2.95. The low D_k_ of pure PA10T may arise from its high free volume and relatively low chain polarity. In addition, polar polymers generally have lower D_k_ at high frequencies than at low frequencies because polar polymers require a certain amount of time to align the dipoles under an alternating electric field [42]. In other words, the dipoles lack sufficient time to align before the field changes direction at very high frequencies. As the PPO content increases, the D_k_ values of PA10T/PPO blends slightly decrease. This is because the non-polar PPO possesses a lower D_k_ than pure PA10T at any frequency. Unlike the D_k_, the D_f_ results from the inability of the polarization process in a molecule to follow the rate of change in the oscillating applied electric field [43]. The current oscillation frequency significantly surpasses the relaxation time of PA10T, resulting in the absorption and dissipation of energy as heat. Therefore, the D_f_ of PA10T at 2.5 GHz is 0.00971 and the values of PA10T/PPO blends decrease rapidly from 0.00866 to 0.00572 as the PPO content increases from 10% to 40%. In fact, PPO has a low D_f_ in board frequency ranges, making it a common choice as a polymer matrix or a crucial component in 5G communication materials.

### 3.6. Mechanical Properties

**Figure 12:**
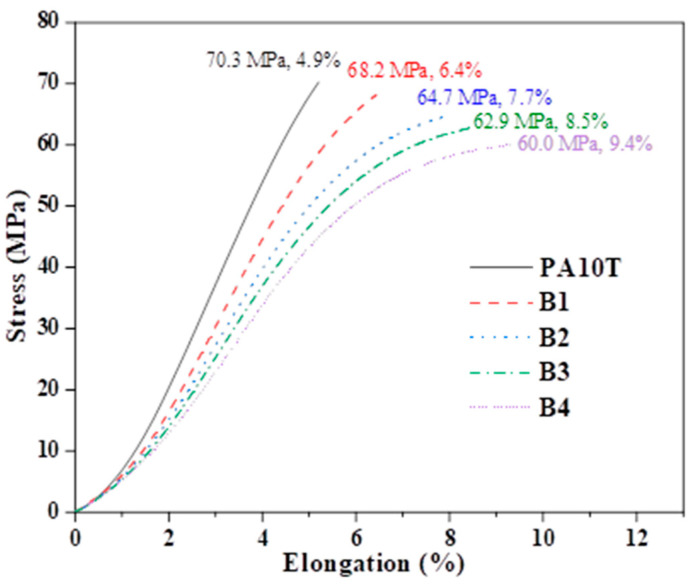
Tension stress–strain of pure PA10T and the PA10T/PPO blends.

The stress–strain curves of pure PA10T and the blends are presented in Figure 12. The summarized mean values for tensile strength, flexural strength, flexural modulus, and notched Izod impact strength are presented in Table 2. The PA10T specimen breaks before reaching the yield stress, elongating at a break as low as 3.9%, indicating its poor flexibility. This finding aligns with previous studies and is primarily attributed to the abundance of stiff benzene rings within its molecular structure [44,45]. The elongation at break for the blends is significantly higher than that of pure PA10T; this result is due to the relatively high flexibility and low shrinkage of the PPO/SEBS-g-MAH components. Such characteristics could reduce residual stress and drawbacks in tensile specimens, thereby preventing the unexpected brittle failure of pure PA10T. The tensile and flexural strengths of PA10T/PPO blends are lower than those of pure PA10T, and these values also decrease with increasing PPO/SEBS-g-MAH content. The inferior strength can be attributed to two main factors: firstly, PA10T has a higher mechanical strength than PPO; secondly, the compatibility between PA10T and PPO is inadequate, even if SEBS-g-MAH partially improves it. As the PPO/SEBS-g-MAH content increases, there is a notable improvement in the toughness of the blends. The notched Izod impact strength for pure PA10T is only 7.9 kJ/m^2^, whereas it increases to 13.7 kJ/m^2^ in the B4 sample. The improvement in toughness is primarily due to the addition of SEBS-g-MAH, which is extensively used not only as a compatibilizer but also as a toughening agent in modified engineering plastics.

## 4. Conclusions

In this work, SEBS-g-MAH, featuring relatively low D_k_ and D_f_, was employed as a commercially available compatibilizer for blending PA10T with PPO. Several blends of PA10T/PPO, with a fixed proportion of compatibilizer (PPO:SEBS-g-MAH = 5:1) and varying PPO contents of 10, 20, 30, and 40 wt%, were prepared and labeled as B1, B2, B3, and B4, respectively. The in situ formation of SEBS-g-PA10T macromolecules resulting from the amidation reaction between PA10T and SEBS-g-MAH serves as an emulsifying agent, effectively stabilizing the PPO particles within the solution or melt of blends. The mean diameter of the PPO phase in B1, B2, B3, and B4 blends is 0.52, 0.47, 1.59, and 1.97 um, respectively. Adding PPO/SEBS-g-MAH to PA10T slightly impacts its thermal stability and crystallization, yet it notably decreases the water absorption and shrinkage rate of PA10T. The D_k_ and D_f_ values of PA10T/PPO blends at 2.5 GHz are significantly reduced compared to those of pure PA10T. The D_f_ of PA10T is 0.00971, while in PA10T/PPO blends, these values rapidly decrease from 0.00866 to 0.00572 when the PPO contents increase from 10% to 40%. Furthermore, SEBS-g-MAH facilitates significant chain extensions and mitigates the melt flow index (MFI) of the blends. The SEBS-g-MAH plays not only a role as a compatibilizer but also as a toughing agent of blends. The B1 blend exhibits the highest tensile strength (68.2 MPa), while B4 demonstrates the highest impact strength (13.7 kJ/m^2^). In conclusion, the developed PA10T/PPO blends exhibit outstanding mechanical and dielectric characteristics, excellent water resistance, low molding shrinkage, and high thermal stability, thereby holding promise for applications in next-generation communication fields.

## Figures and Tables

**Table 1:** The formulations of PA10T and the PA10T/PPO blends.

Designation	Base Resin		Additive SEBS-g-MAH (phr) *
	PA10T (%)	PPO (%)	
PA10T	100	--	--
B1	90	10	2
B2	80	20	4
B3	70	30	6
B4	60	40	8

* phr (parts per hundred) denotes the amount of additives per 100 units of base resin. The SEBS-g-MAH content is maintained at a 1:5 weight ratio to PPO.

**Table 2:** Summary of the mechanical properties of pure PA10T and the PA10T/PPO blends.

Samples	Tensile Strength (MPa)	Flexural Strength (MPa)	Flexural Modulus (MPa)	Notched Izod Impact Strength (kJ/m^2^)
PA10T	70.3	132.3	2670	7.9
B1	68.2	117.0	2430	8.2
B2	64.7	108.7	2310	9.7
B3	62.9	104.4	2220	10.6
B4	60.0	96.7	2040	13.7

## Data Availability

All raw data are available on request.

## References

[1] Jiang J., Wang Z., Sun Y., Qian Z., Cao Z., Wang Z., Zhou G. (2023). Amorphous Poly (Aryl Ether Ketones) Containing Methylene Groups with Excellent Thermal Resistance, Dielectric Properties and Mechanical Performance. Polymers.

[2] Zhang W., Peng Z., Zhang X., Pan Q., Liu S., Cao B., Zhao J. (2022). Soluble liquid crystalline poly(ester imide)s with high glass transition temperatures and improved dielectric properties. ACS Appl. Polym. Mater..

[3] Hasegawa M., Fukuda T., Ishii J. (2024). Poly(ester imide)s with Low Linear Coefficients of Thermal Expansion and Low Water Uptake (VII): A Strategy to Achieve Ultra-Low Dissipation Factors at 10 GHz. Polymers.

[4] Zhu R., Pu Z., He G., Peng Q., Zheng P., Wu F., Yu X., Wang D., Hou H., Li X. (2023). Effect of addition of 1,6-hexanediamine on thermal properties and crystallization behaviors of biobased PA10T polyamides. J. Polym. Res..

[5] Wang W., Wang X., Li R., Liu B., Wang E., Zhang Y. (2009). Environment-friendly synthesis of long chain semiaromatic polyamides with high heat resistance. J. Appl. Polym. Sci..

[6] Shang Y., Lou H., Zhao W., Zhang Y., Cui Z., Fu P., Pang X., Zhang X., Liu M. (2022). The Structural Evolu-tion and Mechanical Properties of Semi-Aromatic Polyamide 12T after Stretching. Polymers.

[7] Zhu R., Pu Z., Zheng L., Zheng P., Wu F., Yu D., Wang X., Yue M., Yang Y., Yang K. (2024). Effect of addition of fillers (MoS2 and PFA) on the tribological and mechanical properties of biobased semi-aromatic polyamide (PA10T). Polym. Compos..

[8] Apeldorn T., Keilholz C., Wolff-Fabris F., Altstädt V. (2013). Dielectric properties of highly filled thermoplastics for printed circuit boards. J. Appl. Polym. Sci..

[9] Zhang L., Chen D., Jin B., Zhang B., Gong P., Zhang B., Park C., Li G. (2023). Ultrahigh electromagnetic wave transmitting polyphenylene sulfide microcellular foams based on molecular structure design for 5G communication. Ind. Eng. Chem. Res..

[10] Liu B., Long J., Chen L., Xiao X., Lin X., Wang Y. (2021). Semi-aromatic polyamides containing fluorenyl pendent toward excellent thermal stability, mechanical properties and dielectric performance. Polymer.

[11] Ning J., Tian C., Yang Y., Huang L., Lv J., Zeng F., Liu Q., Zhao F., Kong W., Cai X. (2021). A novel intrinsic semi-aromatic polyamide dielectric toward excellent thermal stability, mechanical robustness and dielectric performance. Polymer.

[12] Ge Z., Yang S., Tao Z., Liu J., Fan L. (2004). Synthesis and characterization of novel soluble fluorinated aromatic polyamides derived from fluorinated isophthaloyl dichlorides and aromatic diamines. Polymer.

[13] Gnanasekaran D., Ajit Walter P., Reddy B. (2013). Influence of moieties on morphology, thermal, and dielectric properties in polyamide-polyhedral oligomeric silsequioxanes nanocomposites. Polym. Eng. Sci..

[14] Gnanasekaran D., Thomas P. (2021). Enhanced dielectric and thermal performance of bio-polyamide polyhedral oligomeric silsesquioxane (PA-POSS) nanocomposites. IEEE Trans. Dielectr. Electr. Insul..

[15] Luo K., Song G., Wang Y., Yu J., Zhu J., Hu Z. (2019). Low-k and recyclable high-performance POSS/polyamide composites based on Diels−Alder reaction. ACS Appl. Polym. Mater..

[16] Koning C., Martin V., Pagnoulle C., Jerome R. (1998). Strategies for compatibilization of polymer blends. Prog. Polym. Sci..

[17] Zhang Y., Wang M., Zhang D., Wang Y., Wang L., Qiu Y., Wang L., Chen T., Zhao L. (2023). Crystallization and Performance of Polyamide Blends Comprising Polyamide 4, Polyamide 6, and Their Copolymers. Polymers.

[18] Zhang Y., Dai X., Yang D., Guo S., Yang J. (2024). Polyamide 66/poly(2,6-dimethyl-1,4-phenylene oxide) compatibilization with styrene-acrylonitrile-glycidyl methacrylate: Rheology, morphology, and mechanical properties. Int. J. Polym. Anal. Charact..

[19] Guo Z., Shen Y., Fang Z. (2014). Compatibilization of polyamide 6/poly(2,6-dimethyl-1,4-phenylene oxide) blends by poly(styrene-co-maleic anhydride). J. Polym. Eng..

[20] Zhang Z., Cai K., Liu S., Guo W., Zhang B., Yang M., Liu W. (2022). The effect of HIPS-g-MAH on the mechanical properties of PA66/PPO alloy. Polym. Bull..

[21] Jeziorska R., Szadkowska A., Studzinski M. (2022). Morphology and properties of poly(2,6-dimethyl-1,4-phenylene oxide)/polyamide 11 hybrid nanocomposites: Effect of silica surface modification. Materials.

[22] Ai T., Feng W., Ren Z., Li F., Wang P., Zou G., Ji J. (2022). Simultaneous enhancement of mechanical performance and thermal conductivity for polyamide 10T by nanodiamond compositing. J. Appl. Polym. Sci..

[23] Xia H., Jiang Z., Zhou J., Yang Z., Cao Y., Wan Z., Niu J. (2023). Green synthesis of reactive copolymers in molten ε-caprolactam solvent and their compatibilizing effects in PA10T/PPO blends. J. Appl. Polym. Sci..

[24] Wang S., Li B., Zhang Y. (2010). Reactive compatibilisation and toughening of poly(2,6-dimethyl-1,4-phenylene oxide)/polyamide 6 blends by maleic grafted styrene ethylene butadiene styrene copolymer and styrene maleic anhydride copolymer. Plast. Rubber Compos..

[25] Li B., Wan C., Zhang Y., Ji J., Su Y. (2010). Reactive compatibilization and elastomer toughening of poly(2,6-dimethyl-1,4-phenylene oxide)/polyamide 6 blends. Polym. Polym. Compos..

[26] Ryu Y., Sohn J., Yun C., Cha S. (2020). Shrinkage and warpage minimization of glass-fiber-reinforced polyamide 6 parts by microcellular foam injection molding. Polymers.

[27] (2008). Plastics—Determination of Water Absorption.

[28] Aparna S., Purnima D., Adusumalli R. (2020). Effect of short carbon fiber content and water absorption on tensile and impact properties of PA6/PP blend based composites. Polym. Compos..

[29] (2012). Plastics—Determination of Tensile Properties.

[30] (2019). Plastics—Determination of Flexural Properties.

[31] (2019). Plastics—Determination of Izod Impact Strength.

[32] Zhang Z., Yang M., Cai K., Chen Y., Liu S., Liu W., Liu J. (2022). Effect of the flame retardants and glass fiber on the polyamide 66/polyphenylene oxide composites. Materials.

[33] Yang C., Huang Y., Dong J., Li N. (2019). Anion-conductive poly(2,6-dimethyl-1,4-phenylene oxide) grafted with tailored polystyrene chains for alkaline fuel cells. J. Membr. Sci..

[34] Wang S., Li B., Zhang Y. (2010). Compatibilization of poly(2,6-dimethyl-1,4-phenylene oxide)/polyamide 6 blends with styrene-maleic anhydride copolymer: Mechanical properties, morphology, crystallization, and melting behavior. J. Appl. Polym. Sci..

[35] Luna C., Filho E., Siqueira D., Araújo E., Nascimento E., Melo T. (2022). Influence of small amounts of ABS and ABS-MA on PA6 properties: Evaluation of torque rheometry, mechanical, thermomechanical, thermal, morphological, and water absorption kinetics characteristics. Materials.

[36] Li Y., Shimizu H. (2004). Novel morphologies of poly(phenylene oxide) (PPO)/polyamide 6 (PA6) blend nanocomposites. Polymer.

[37] Nakano A., Kubota Y., Osaka N., Higashimura H. (2022). Enzyme model-catalyzed, oxidative copolymerization of phenol while continuously adding an endcap to multi-branched poly(phenylene oxide) showing low dielectric constant. Chem. Lett..

[38] Zhu W., Weil E. (1998). Influence of the molecular weight of PPO resins and char-forming behavior of polymeric additives on the flame retardancy of EPDM formulations. J. Appl. Polym. Sci..

[39] Li M. (2019). Study on melting and polymorphic behavior of poly(decamethylene terephthalamide). J. Appl. Polym. Sci..

[40] Yang K., Xin C., Huang Y., Jiang L., He Y. (2017). Effects of extensional flow on properties of polyamide-66/poly(2,6-dimethyl-1,4-phenylene oxide) blends: A study of morphology, mechanical properties, and rheology. Polym. Eng. Sci..

[41] Capone C., Di Landro L., Inzoli F., Penco M., Sartore L. (2007). Thermal, and mechanical degradation during polymer extrusion processing. Polym. Eng. Sci..

[42] Pekdemir M., Tukur A., Coskun M. (2021). Thermal and dielectric investigation of magnetic nanoparticles functionalized with PVC via click chemistry. Polym. Bull..

[43] Rosa A., Ursan G., Aradoaei M., Ursan M., Schreiner C. Susceptor assisted microwave processing of polymers for adhesive production. Proceedings of the 2018 International Conference and Exposition on Electrical And Power Engineering (EPE).

[44] Li M., Bijleveld J., Dingemans T. (2018). Synthesis and properties of semi-crystalline poly(decamethylene terephthalamide) thermosets from reactive side-group copolyamides. Eur. Polym. J..

[45] Ai T., Feng W., Zou G., Ren Z., Wang P., Ji J., Zhang W. (2020). High-performances biobased semi-aromatic polyamide 10T copolymerized with silicone monomers. J. Appl. Polym. Sci..

